# Whole Brain Functional Connectivity Pattern Homogeneity Mapping

**DOI:** 10.3389/fnhum.2018.00164

**Published:** 2018-04-24

**Authors:** Lijie Wang, Jinping Xu, Chao Wang, Jiaojian Wang

**Affiliations:** ^1^The Clinical Hospital of Chengdu Brain Science Institute, MOE Key Lab for Neuroinformation, University of Electronic Science and Technology of China, Chengdu, China; ^2^School of Life Science and Technology, Center for Information in Medicine, University of Electronic Science and Technology of China, Chengdu, China; ^3^Institute of Biomedical and Health Engineering, Shenzhen Institutes of Advanced Technology, Chinese Academy of Sciences, Shenzhen, China; ^4^College of Psychology and Sociology, Shenzhen University, Shenzhen, China

**Keywords:** whole brain functional connectivity, similarity, Kendall’s coefficient concordance, fMRI, voxel-wise

## Abstract

Mounting studies have demonstrated that brain functions are determined by its external functional connectivity patterns. However, how to characterize the voxel-wise similarity of whole brain functional connectivity pattern is still largely unknown. In this study, we introduced a new method called functional connectivity homogeneity (FcHo) to delineate the voxel-wise similarity of whole brain functional connectivity patterns. FcHo was defined by measuring the whole brain functional connectivity patterns similarity of a given voxel with its nearest 26 neighbors using Kendall’s coefficient concordance (KCC). The robustness of this method was tested in four independent datasets selected from a large repository of MRI. Furthermore, FcHo mapping results were further validated using the nearest 18 and six neighbors and intra-subject reproducibility with each subject scanned two times. We also compared FcHo distribution patterns with local regional homogeneity (ReHo) to identify the similarity and differences of the two methods. Finally, FcHo method was used to identify the differences of whole brain functional connectivity patterns between professional Chinese chess players and novices to test its application. FcHo mapping consistently revealed that the high FcHo was mainly distributed in association cortex including parietal lobe, frontal lobe, occipital lobe and default mode network (DMN) related areas, whereas the low FcHo was mainly found in unimodal cortex including primary visual cortex, sensorimotor cortex, paracentral lobule and supplementary motor area. These results were further supported by analyses of the nearest 18 and six neighbors and intra-subject similarity. Moreover, FcHo showed both similar and different whole brain distribution patterns compared to ReHo. Finally, we demonstrated that FcHo can effectively identify the whole brain functional connectivity pattern differences between professional Chinese chess players and novices. Our findings indicated that FcHo is a reliable method to delineate the whole brain functional connectivity pattern similarity and may provide a new way to study the functional organization and to reveal neuropathological basis for brain disorders.

## Introduction

Resting-state functional magnetic resonance imaging (rs-fMRI) which primarily reflects the ongoing spontaneous fluctuations in the human brain is a task-independent approach to detect intrinsic neural activity related to self-initiated behavior (Fox and Raichle, 2007). By measuring the low-frequency blood oxygen level dependent (BOLD) signal, rs-fMRI has been widely used to explore the functional coupling between brain areas (Biswal et al., 1995; Wang et al., 2016a, 2017a, 2018a,b; Wu H. et al., 2016; Wu et al., 2016b). A large number of studies have documented that brain functions were determined by its external connectivity patters, i.e., connectivity fingerprints (Passingham et al., 2002; Toro et al., 2008; Wang et al., 2012, 2015a,b; Wu et al., 2016a; Yang et al., 2016; Zhang et al., 2016). Recently, based on different whole brain functional connectivity patterns, many researches were performed to parcellate the human brain areas into different functional subareas (Yeo et al., 2011; Wang et al., 2015b, 2016b, 2017b).

Several data-driven methods have been proposed to characterize the functional activities similarity. Li et al. (2002) proposed cross-correlation coefficients of spontaneous low frequency (COSLOF) to measure the functional synchrony between possible pairs of voxel time courses in a brain region. Subsequently, Zang et al. (2004) developed a regional homogeneity (ReHo) method to measure the similarity of the time series of a given voxel and its nearest 26 neighbors using Kendall’s coefficient concordance (KCC). Additionally, Deshpande et al. (2009) introduced a measure of integrated local correlation (ILC) for assessing local coherence in the brain. Recently, Tomasi and Volkow (2010) proposed functional connectivity density (FCD) to further characterize regionally functional homogeneity in the human brain. Although different measurements have been proposed to characterize the similarities of functional activities and regionally functional connectivities, how to map the voxel-wise whole brain functional connectivity pattern similarity is still largely unknown. To map voxel-wise whole brain functional connectivity patterns similarity will provide an important approach to investigate the functional architecture of the brain.

In the present study, we introduced a voxel-wise manner to characterize the whole brain functional connectivity homogeneity (FcHo) for a specific voxel with its nearest 26 voxels in four independent rs-fMRI datasets using KCC. We validated this method by measuring the similarity of a specific voxel with its nearest 18 and 6 neighbors and intra-subject reproducibility. FcHo was also compared to ReHo to reveal the similarity and differences of the two methods and was applied to identify the functional connectivity patterns differences between professional Chinese chess players and novice to test its applicability.

## Materials and Methods

### Subjects

rs-fMRI datasets in this study were accessed from the “1000 Functional Connectomes’ Project”^1^. Four independent datasets (Berlin, Leipzig, Dallas and Newark) including 106 subjects were selected and used for whole brain FcHo mapping. Berlin dataset includes 26 participants (13 females and 13 males) with age ranging from 23 years to 44 years (mean age = 29.77 years, SD = 5.21). For Dallas dataset, 24 participants including 12 females and 12 males with age ranging from 20 years to 71 years (mean age = 42.63 years, SD = 20.07) were used for FcHo mapping. For Leipzig dataset, 37 participants include 21 females and 16 males with age ranging from 20 years to 42 years (mean age = 26.22 years, SD = 5.01). For Newark dataset, 19 participants including 10 females and 9 males with age ranging from 21 years to 39 years (mean age = 24.11 years, SD = 3.91) were used in this study. To further validate the reliability of FcHo method, 25 subjects’ fMRI datasets (10 males and 15 females with age ranging from 22 years to 49 years, mean age = 29.44 years, SD = 8.64) were also downloaded from 1000 Functional Connectomes’ Project (NewYork_Test-Retest_Reliability), and each subject in this dataset was scanned two times with interval of 5–11 months after the first resting-state scan (Zuo et al., 2010). To explore whether FcHo is an effective method to detect the group differences, we applied FcHo method to study 29 professional Chinese chess players (20 females and 9 males, age ranging from 15 years to 59 years, mean age = 28.72 years, SD = 10.84) and 29 age and sexually well-matched novices (15 females and 14 males, age ranging from 17 years to 43 years, mean age = 25.76 years, SD = 6.95) to reveal the differences in whole brain functional connectivity patterns. This set of fMRI data was also downloaded from 1000 Functional Connectomes’ Project (Huaxi MR Research Center (HMRRC), West China Hospital of Sichuan University).

### Resting-State fMRI Data Preprocessing

The rs-fMRI data was preprocessed using SPM8 software^2^. The preprocessing steps includes: discarding the first 10 volumes to allow for magnetization equilibrium, head motion correction by realigning to the first volume (a maximum displacement in any of the cardinal directions >3 mm, or a maximum spin >3° were excluded, under this criterion, no subjects excluded), normalized to the Montreal Neurological Institute (MNI) EPI template and resampled at 5 × 5 × 5 mm^3^, Friston 24-parameter model of head motion, white matter and cerebrospinal fluid mean signals were regressed out and filtered with a temporal band-path of 0.01–0.1 Hz. To further exclude the head motion effects on functional connectivity analysis, scrubbing method was conducted. Each subject’s fMRI time series were censored to find the mean frame displacement (FD) was above 0.5 mm, and one volume before and two volumes after the bad volume were discarded (Power et al., 2012). Given that global mean signal of the brain affects functional connectivity analyses, we also studied the global signal regression (GSR) on analytical results to further validate our major findings.

### FcHo Mapping

FcHo was measured using KCC (Kendall and Gibbons, 1990). To calculate FcHo of a given voxel, a KCC value was assigned to this voxel by computing the KCC of the whole brain functional connectivity of this voxel with those of its nearest 26 neighbors. After obtained the FcHo map for each subject, one-sample *t*-tests with age and gender as covariates were used to delineate the FcHo distribution in the whole brain by compared to whole brain mean FcHo values.

$$KCC\;=\;\frac{\sum {\left(R_{\text{i}}\right)}^{2}-K{\left(\bar{R}\right)}^{2}}{\frac{1}{12}N^{2}(K^{3}-K)}$$

where *R*_i_ is the sum rank of the *i*th voxel of the whole brain; $\bar{R}\;=\;((K+1)\times N)/2$ is the mean of the *R*_i_; *N* is the number of a given voxel and its nearest neighbors (*N* = 26); *K* is the number of whole brain voxels. KCC among given voxels ranged from 0 to 1.

### Global Signal Regression Effects

Because whole brain GSR affects the functional connectivity mapping, we also calculated the voxel-wise FcHo using fMRI data with GSR using the same procedures to evaluate the reproducibility of our method. To quantify the spatial distribution similarity of FcHo maps calculated using fMRI data with GSR and without GSR (NoGSR), spatial correlation coefficients between the statistical FcHo maps, obtained using one-sample *t*-tests as described above, were calculated in all the four datasets. Moreover, we also measured the spatial distribution similarity across different datasets for FcHo maps calculated using GSR and NoGSR fMRI data. The spatial correlation coefficients between FcHo maps between any pair of datasets were calculated.

### Different Nearest Neighborhood Voxels Reproducibility

In addition, to explore whether the selection of nearest neighbors (*N* = 6 and 18) affects FcHo mapping results, we calculated the voxel-wise FcHo map with the nearest six and 18 voxels using the same procedures. For cross-validation, the FcHo maps under six and 18 voxels were calculated using both GSR and NoGSR fMRI data. The spatial correlation coefficients between FcHo maps obtained with 26 nearest neighborhood voxels and that obtained with 18 and six nearest neighborhood voxels were calculated.

### Intra-Subject Reproducibility

To further explore the stability of FcHo method, the intra-subject FcHo maps were calculated and compared between different scan sessions. The spatial correlation between FcHo distribution patterns of different scan sessions was calculated. Furthermore, the spatial correlation between FcHo distribution patterns obtained using NoGSR and GSR fMRI data was also computed to delineate the similarity of FcHo maps.

### Compared With Regional Homogeneity

Given that ReHo also uses KCC to evaluate the similarity or synchronization between the time series of a given voxel and its nearest 26 neighbors (Zang et al., 2004), we compared our FcHo maps with ReHo maps to reveal the similarity and differences between the two methods and to further validate the reliability of FcHo method.

### FcHo Application

To test applicability of FcHo method, the FcHo maps were calculated for both professional Chinese chess player and novice. Two-sample *t*-test was performed to identify FcHo differences. The significance was set at *P* < 0.01 with cluster size > 10 (uncorrected).

## Results

### FcHo Mapping Results

**Figure 1:**
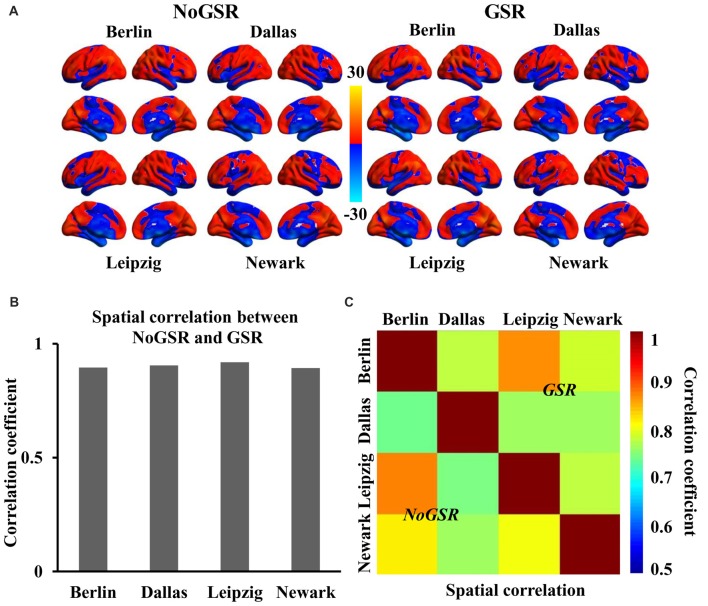
Similar spatial distribution of whole brain functional connectivity homogeneity (FcHo) mapping in four independent datasets (Berlin, Leipzig, Dallas, Newark). **(A)** Voxel-wise FcHo was calculated using functional magnetic resonance imaging (fMRI) data with and without global signal regression (GSR and NoGSR). One-sample *t*-tests with gender and age as covariates were used to identify the spatial distribution of FcHo by compared to whole brain mean FcHo value. **(B)** Spatial correlation coefficients were calculated between FcHo maps obtained using NoGSR and GSR fMRI data in all the four datasets. **(C)** Spatial correlation coefficients were also calculated between any pair of FcHo maps across all the four datasets.

Voxel-wise FcHo mapping results based on 26 nearest neighbors in four independent datasets were showed in Figure 1. The higher FcHo values than whole brain mean was mainly observed in default mode network (DMN), parietal lobe (superior/inferior parietal lobule and precuneus), lateral prefrontal cortex, dorsomedial prefrontal cortex, occipital cortex, cuneus, and dorsal anterior insula (Figure 1A). The lower FcHo was primarily found in sensorimotor cortex, paracentral lobule, medial frontal cortex/supplementary motor area, cingulate motor area, medial temporal lobule and ventral anterior and posterior insula (Figure 1A).

To validate whether FcHo mapping results were affected by whole brain GSR, the spatial correlation analyses revealed that the whole brain FcHo maps obtained using fMRI data with and without GSR showed similar distribution patterns (*R*_Berlin_ = 0.895, *R*_Dallas_ = 0.905, *R*_Leipzig_ = 0.919, *R*_Newark_ = 0.894, all *P* ≈ 0; Figure 1B). Moreover, the spatial correlation analyses across different datasets also revealed the high spatial overlap of FcHo patterns obtained using NoGSR (*R*_Berlin & Dallas_ = 0.741, *R*_Berlin & Leipzig_ = 0.875, *R*_Berlin & Newark_ = 0.815, *R*_Dallas & Leipzig_ = 0.743, *R*_Dallas & Newark_ = 0.761, *R*_Leipzig & Newark_ = 0.799, all *P* ≈ 0) and GSR (*R*_Berlin & Dallas_ = 0.777, *R*_Berlin & Leipzig_ = 0.866, *R*_Berlin & Newark_ = 0.788, *R*_Dallas & Leipzig_ = 0.764, *R*_Dallas & Newark_ = 0.763, *R*_Leipzig & Newark_ = 0.78, all *P* ≈ 0) fMRI data (Figure 1C).

### Different Nearest Neighborhood Voxels Reproducibility

**Figure 2:**
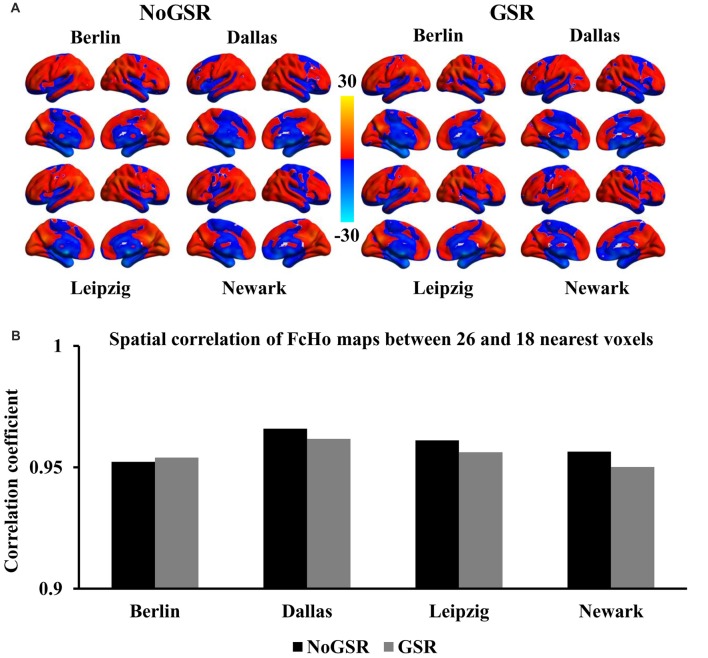
Whole brain FcHo mapping using the nearest 18 neighbors. **(A)** To test the selection of number of nearest voxels, FcHo maps were calculated using the nearest 18 neighborhood voxels using fMRI data with and without GSR (GSR and NoGSR), and the spatial distribution patterns were similar with the FcHo maps obtained using 26 nearest voxels. **(B)** Spatial correlation coefficients were separately calculated between FcHo maps obtained with 26 and 18 nearest voxels by using NoGSR and GSR fMRI data in all the four datasets.

**Figure 3:**
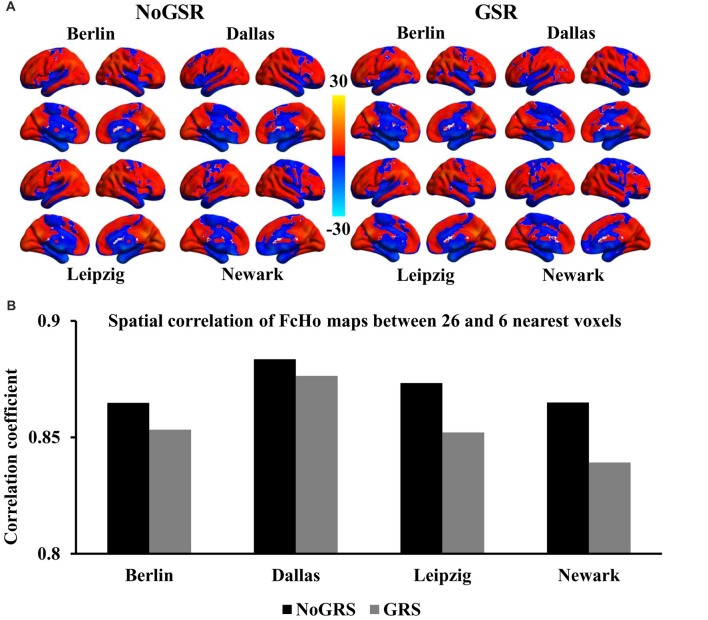
Whole brain FcHo mapping using the nearest six neighbors. **(A)** FcHo maps were also calculated using the nearest six neighborhood voxels using fMRI data with and without GSR (GSR and NoGSR) to test the effect nearest voxels, and similar spatial distribution patterns were obtained. **(B)** Spatial correlation coefficients were calculated between FcHo maps obtained with 26 and six nearest voxels by using NoGSR and GSR fMRI data in all the four datasets, respectively.

To further validate whether the voxel-wise FcHo mapping was affected by the selection of the number of nearest neighbors, FcHo mapping using 18 and 6 nearest neighbors were also calculated. The spatial distribution patterns calculated using nearest 18 and six voxels were similar with that obtained with nearest 26 voxels (Figures 2A, 3A). The spatial correlations between the FcHo maps with 26 nearest voxels and 18 nearest voxels (*R*_Berlin(NoGSR)_ = 0.952, *R*_Berlin(GSR)_ = 0.954, *R*_Dallas(NoGSR)_ = 0.966, *R*_Dallas(GSR)_ = 0.962, *R*_Leipzig(NoGSR)_ = 0.961, *R*_Leipzig(GSR)_ = 0.956, *R*_Newark(NoGSR)_ = 0.956, *R*_Newark(GSR)_ = 0.95, all *P* ≈ 0), six nearest voxels (*R*_Berlin(NoGSR)_ = 0.865, *R*_Berlin(GSR)_ = 0.853, *R*_Dallas(NoGSR)_ = 0.884, *R*_Dallas(GSR)_ = 0.876, *R*_Leipzig(NoGSR)_ = 0.873, *R*_Leipzig(GSR)_ = 0.852, *R*_Newark(NoGSR)_ = 0.865, *R*_Newark(GSR)_ = 0.839, all *P* ≈ 0) also showed high consistency (Figures 2B, 3B).

### Intra-Subject Reproducibility

**Figure 4:**
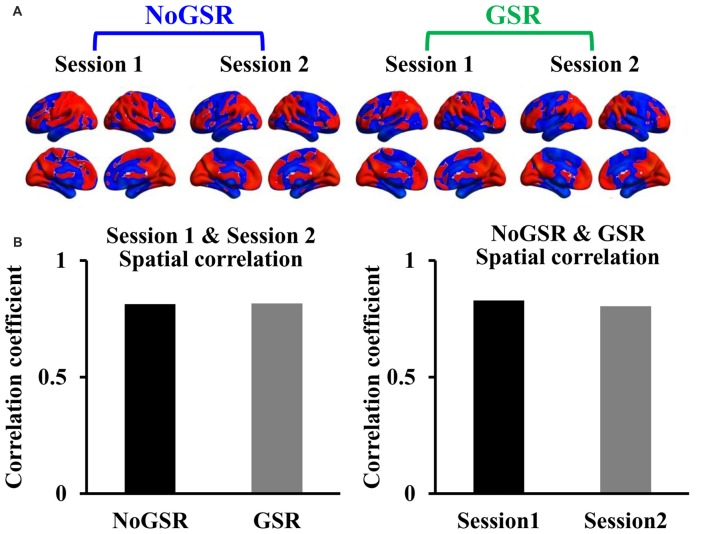
Intra-subject similarity of whole brain FcHo mapping using the nearest 26 neighbors. **(A)** To test the stability of our method, the intra-subject similarity of FcHo maps was measured by using the same subject scanned two times fMRI data with and without GSR (GSR and NoGSR). The spatial distribution patterns of FcHo were similar between different scanning sessions and between FcHo maps obtained with NoGSR and GSR fMRI data. **(B)** Quantitative spatial correlation analyses also identified high correlation coefficients between different sessions and between different preprocessing methods.

The intra-subject spatial distribution patterns of FcHo maps showed high similarity between different scan sessions and between maps calculated using fMRI data with and without GSR (Figure 4A). The quantitative spatial correlation analyses between different sessions (*R*_Session1 & Session2 (NoGSR)_ = 0.814, *R*_Session1 & Session2 (GSR)_ = 0.817, all *P* ≈ 0) and between FcHo maps calculated using GSR and NoGSR (*R*_NoGSR & GSR (Session1)_ = 0.83, *R*_NoGSR & GSR (Session2)_ = 0.805, all *P* ≈ 0) also identified consistent whole brain FcHo patterns (Figure 4B).

### Compared with ReHo

**Figure 5:**
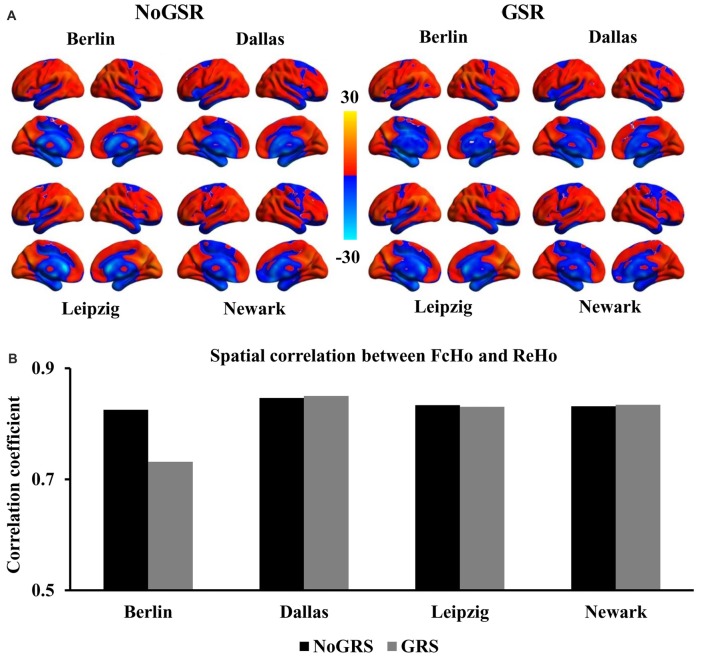
Comparisons of whole brain FcHo mapping and local regional homogeneity (ReHo) mapping using the nearest 26 neighbors. **(A)** ReHo maps were calculated using the nearest 26 neighborhood voxels using fMRI data with and without GSR (GSR and NoGSR), and whole brain spatial distribution patterns were obtained. **(B)** Spatial correlation coefficients between FcHo and ReHo maps were calculated using both NoGSR and GSR fMRI data in all the four datasets showing similar spatial distribution patterns.

**Figure 6:**
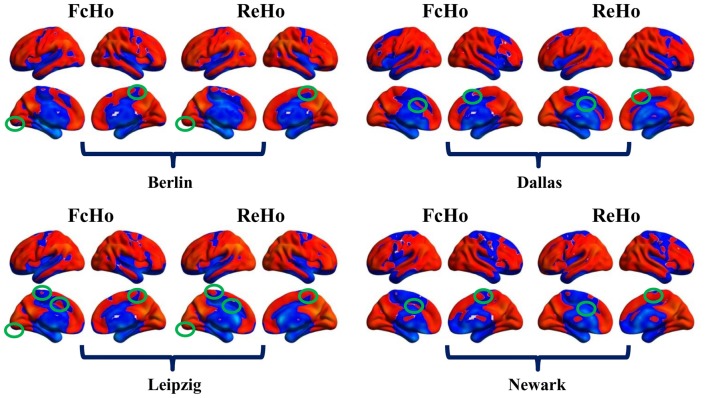
Differences between whole brain FcHo and local ReHo mapping using the nearest 26 neighbors. Besides the similar distribution patterns between FcHo and ReHo, we also identified different distribution patters in primary visual cortex, paracentral lobule, supplementary motor area and middle cingulate cortex. FcHo method more sensitively identified lower FcHo values in primary cortex (such as visual cortex, paracentral lobule and supplementary motor area in this study) and higher FcHo values in association cortex (such as middle cingulate cortex in this study) than ReHo method.

Because of both FcHo and ReHo using KCC to delineate the similarity, we compared the spatial distribution of FcHo and ReHo patterns in the four different datasets. Whole brain spatial distribution patterns of ReHo were similar with FcHo (Figure 5A), which was further demonstrated by the following spatial correlation analyses (*R*_Berlin(NoGSR)_ = 0.825, *R*_Berlin(GSR)_ = 0.732, *R*_Dallas(NoGSR)_ = 0.846, *R*_Dallas(GSR)_ = 0.85, *R*_Leipzig(NoGSR)_ = 0.834, *R*_Leipzig(GSR)_ = 0.831, *R*_Newark(NoGSR)_ = 0.832, *R*_Newark(GSR)_ = 0.834, all *P* ≈ 0; Figure 5B). We also found differences in spatial distribution patterns between FcHo and ReHo maps in the four datasets (Figure 6). The main differences were mainly found in paracentral lobule, primary visual cortex, supplementary motor area and middle cingulate cortex. Our method identified low FcHo patterns in unimodal areas including paracentral lobule, primary visual cortex, and supplementary motor area and high FcHo patterns in middle cingulate cortex which is a part of association cortex.

### FcHo Application

**Figure 7:**
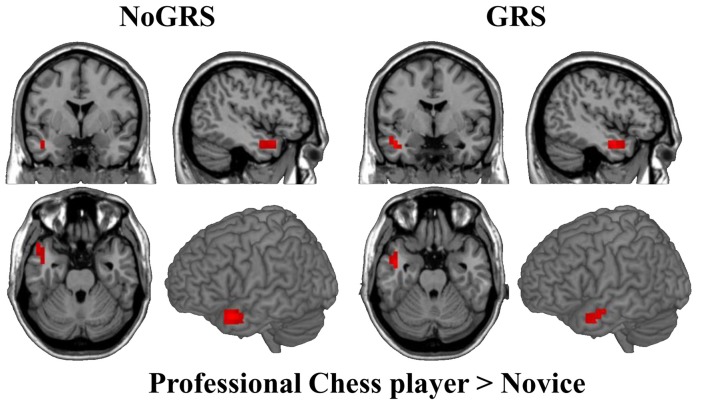
Differences in whole brain FcHo between professional Chinese chess player and novice. Two-sample *t*-test was used to identify FcHo differences between professional Chinese chess player and novice. Higher FcHo in anterior middle temporal gyrus (MTG) was found in professional Chinese chess player compared to novice (*P* < 0.01, cluster size >10, uncorrected).

To test whether FcHo is a reliable method to reveal changed whole brain functional connectivity patterns, two-sample *t*-test was performed between professional Chinese chess players and novices. Statistical analysis identified significant increased FcHo values in anterior middle temporal gyrus (MTG) using both NoGSR and GSR fMRI data (Figure 7).

## Discussion

In this study, a data-driven method was introduced to map the voxel-wise whole brain functional connectivity pattern similarity, i.e., whole brain FcHo, using rs-fMRI. This method was further validated using fMRI data with GSR, different nearest neighbors, intra-subject repetitive scanning, comparison with ReHo, and application to identify functional connectivity pattern differences between professional Chinese chess player and novice. Our findings demonstrated that FcHo was a reliable method to map the whole brain functional connectivity pattern similarity and may facilitate the future cognitive and clinical studies.

In this study, we used KCC to measure the whole brain functional connectivity patterns similarity for a given voxel with its 26 nearest neighbors. To measure the voxel-wise FcHo, KCC has many advantages. First, KCC is a rank-based non-parametric data-driven approach and is more robust against noise. Second, KCC does not need the prior knowledge and is free of parametric settings to study functional similarity (Zuo et al., 2013). Using the same measurement, Zang et al. (2004) developed a local ReHo method to delineate functional signal similarity. Thus, KCC is a good choice to characterize similarity of whole brain functional connectivity patterns.

Several different methods have been proposed to characterize the functional similarity, but none of these methods have directly explored to delineate the voxel-wise whole brain functional connectivity similarity. Zang et al. (2004) used KCC to calculate the voxel-wise ReHo of voxels’ time series. However, whether the similarity of time series can reflect the whole brain functional connectivity pattern similarity needs to be further confirmed. A recent study developed FCD method to delineate the functional connectivity similarity with its neighborhoods using regional growing approach (Tomasi and Volkow, 2010), however, how to select the thresholds of connectivity strength for calculating FCD is a problem. Furthermore, FCD does not measure the whole brain functional connectivity similarity. In our study, we introduced FcHo method inspired by ReHo mapping. FcHo is a free of threshold setting approach, which may better delineate the whole brain functional connectivity similarity.

The spatial distribution of high FcHo is predominantly localized in DMN, parietal lobe (superior/inferior parietal lobule and precuneus), lateral prefrontal cortex, dorsomedial prefrontal cortex, and cuneus, whereas low FcHo is primarily found in sensorimotor cortex, paracentral lobule, and medial frontal cortex/supplementary motor area. These findings showed that association cortex and high-order cognition related brain areas have higher FcHo values while primary sensory and motor related areas have lower FcHo values. Our findings were supported by the previous FCD mapping results which identified spatial distribution of the local FCD is highly localized in the brain areas showing higher FcHo in our study (Tomasi and Volkow, 2010). In addition, the previous studies have demonstrated that primary sensory and motor areas are heavily myelinated and are rich in local axonal ramifications of pyramidal neurons (Sepulcre et al., 2010; Glasser and Van Essen, 2011). Thus, the low FcHo in primary sensory and motor areas may result from the dominant local short-range connections (Wang et al., 2017c).

We identified increased FcHo in anterior MTG in professional Chinese chess players compared to novices. This finding was supported by a previous study which used the same participants and found significant deactivation in this area in professional Chinese chess players during task (Duan et al., 2012). The previous resting-state functional connectivity analyses revealed that the anterior MTG mainly connected with DMN related areas (Buckner et al., 2008; Xu et al., 2015). Thus, anterior MTG is an important part of DMN suggesting that anterior MTG may play an important role in episodic memory (Buckner et al., 2008). Moreover, a task-related fMRI study has revealed that anterior MTG was related to semantic retrieval (Cho et al., 2012). Thus, we speculated that higher FcHo in anterior MTG found in professional chess player may suggest better integration of semantic and episodic processing than novice.

There are several limitations in the current study. During fMRI data preprocessing, we normalized fMRI data to the MNI template space and resampled at 5 × 5 × 5 mm^3^ because of the computation capability constrain in our lab. When we calculated the voxel-wise FcHo using the resolution of cubic 3 or 4 mm, the computing speed is very slow. Thus, the voxel-wise resolution of cubic 5 mm was finally adopted. This resolution is enough to map the spatial distribution, but is a little lower in clinical studies. Thus, the cortical surface or cortical skeleton masks will facilitate the future clinical studies using this method. Second, although we used four independent datasets to validate our method, the number of subjects in each dataset is very small. Our findings need to be further validated using a larger sample dataset.

## Author Contributions

LW and JW designed research, performed research, analyzed data and wrote the article. JX and CW contributed to the discussions and revisions.

## Conflict of Interest Statement

The authors declare that the research was conducted in the absence of any commercial or financial relationships that could be construed as a potential conflict of interest.

## Footnotes

^1^http://www.nitrc.org/projects/fcon_1000/

^2^http://www.fil.ion.ucl.ac.uk/spm
